# Perceived stress and high-sensitivity C-reactive protein among healthcare workers: A cross-sectional analysis

**DOI:** 10.1192/j.eurpsy.2026.12104

**Published:** 2026-07-31

**Authors:** F. Maher, M. Bouhoula, S. Mrad, O. Salah, M. Makhloufi, I. Kacem, A. Aloui, A. Chouchane, H. Kalboussi, O. El Maalel, S. Chatti, B. Charfeddine, M. Maoua

**Affiliations:** 1 Department of Biochemistry, Farhat Hached University Hospital, Sousse, Tunisia; 2 Department of Occupational Medicine, Farhat Hached University Hospital, Research Laboratory LR19SP03, Sousse, Tunisia, University of Sousse, Faculty of Medicine of Sousse, Sousse, Tunisia, Sousse, Tunisia

## Abstract

**Introduction:**

Psychosocial stress may upregulate low-grade inflammation implicated in atherosclerosis. High-sensitivity C-reactive protein (hs-CRP) is a validated biomarker of cardiovascular inflammatory risk (low <1 mg/L, average 1–3 mg/L, high >3 mg/L). The Perceived Stress Scale (PSS-10) offers a quantification of stress in clinical-epidemiological settings.

**Objectives:**

To evaluate the association between perceived stress levels and High-sensitivity C-reactive protein value among healthcare professionals

**Methods:**

We conducted a cross-sectional analytic study among hospital personnel at CHU Farhat Hached (Sousse, Tunisia). Perceived stress was assessed with the 10-item Perceived stress Score **(**PSS-10). Fasting blood (≥12 h) was analyzed for hs-CRP in the biochemistry laboratory. Associations between stress and hs-CRP were explored using Spearman correlation and Mann–Whitney tests, following the predefined analysis plan.

**Results:**

Among participants with available hs-CRP (n=9 of 133 with PSS-10), mean age was 43.8±9.9 years; 8/9 were women; mean BMI 26.9±3.8 kg/m². Median hs-CRP was 2.0 mg/L (IQR 1–3); 2/9 (22.2%) were in the high-risk category (>3 mg/L), and 7/9 (77.8%) in the average-risk category (1–3 mg/L). One value exceeded 10 mg/L (20 mg/L), a level for which retesting to exclude acute inflammation is recommended. PSS-10 averaged 20.1±3.8; most participants were in the moderate stress range (8/9), with 1/9 low and none high. Stress and hs-CRP showed a positive but non-significant correlation (Spearman ρ=0.29; p=0.44). Median hs-CRP was 1.0 mg/L in the low-stress group vs 2.0 mg/L in the moderate-stress group (Mann–Whitney p=0.42). In a sensitivity analysis excluding the single value >10 mg/L, the correlation remained non-significant (ρ=0.35; p=0.39).

**Conclusions:**

In this preliminary subset with hs-CRP, perceived stress trended positively—but not significantly—with hs-CRP. Most values fell in the “average-risk” range, with a minority >3 mg/L. These early findings mirror the mixed literature on the stress–CRP link and highlight the need for larger samples and careful handling of potential acute inflammatory outliers when evaluating low-grade inflammation in shift-working healthcare staff.

**Disclosure of Interest:**

None Declared

